# Impact of a Single Hemodialysis Session on Oxidative Stress-Inducing and Oxidative Damage Biomarkers in End-Stage Kidney Disease Patients

**DOI:** 10.3390/cimb48050482

**Published:** 2026-05-06

**Authors:** Athina Varemmenou, Effimia Michail, Electra Kalaitzopoulou, Polyxeni Papadea, Marianna Skipitari, Marios Papasotiriou, Evangelos Papachristou, Dimitrios Goumenos, Christos D. Georgiou

**Affiliations:** 1 Department of Medicine, University of Patras, 265 04 Patras, Greece; athina.var97@gmail.com; 2 Department of Biology, University of Patras, 265 04 Patras, Greece; effie.mich@gmail.com (E.M.); e.kalaitzopoulou@hotmail.com (E.K.); papadeaxenia@gmail.com (P.P.); marianna.sk.14@gmail.com (M.S.); 3 Department of Nephrology and Kidney Transplantation, School of Medicine, University of Patras, 265 04 Patras, Greece; epapachr@upatras.gr (E.P.); dgoumenos@upatras.gr (D.G.)

**Keywords:** chronic kidney disease, hemodialysis, lipid peroxidation, oxidative stress, reactive oxygen species

## Abstract

Oxidative stress (OS) is elevated in patients with end-stage kidney disease undergoing maintenance dialysis and contributes to increased cardiovascular risk. While kidney dysfunction and dialysis can generate OS, the acute effects of a single dialysis session remain unclear due to variability in study design and the biomarkers used. In this observational study, blood samples from 68 hemodialysis patients were collected before and after a single session. Plasma levels of the reactive oxygen species marker superoxide (O_2_^•−^) and OS-damage marker lipid hydroperoxides (LOOHs), protein-bound malondialdehyde (PrMDA), protein-bound thiobarbituric acid reactive substances (PrTBARSs), and protein carbonyls (PrCOs) were measured. LOOHs increased significantly by 50% post-dialysis, whereas PrMDA and PrTBARSs decreased modestly by ~10%. No significant changes were observed in O_2_^•−^ or PrCOs. Dialysis vintage correlated positively with LOOHs, PrMDA, and PrTBARSs, but not with O_2_^•−^ or PrCOs. No significant associations were found between OS markers and comorbidities, medication or sex. The post-dialysis rise in LOOHs, an early-formed and least accumulating lipid peroxidation marker, may reflect acute changes in OS during a single HD session. The rising association of PrMDA and PrTBARSs with dialysis vintage may suggest cumulative OS over time.

## 1. Introduction

Oxidative stress (OS) has emerged as an important risk factor for chronic kidney disease (CKD) and particularly in patients on maintenance hemodialysis (HD). Reactive oxygen species (ROS) are generated under physiological conditions due to aerobic metabolism. However, OS occurs when the production of ROS exceeds their neutralization by endogenous antioxidant defenses [1]. Consequently, DNA, lipids and proteins undergo oxidative damage, impairing cellular and organismal function [2].

Among the most important ROS involved in oxidative damage are the superoxide (O_2_^•−^) and the hydroxyl radical (^•^OH). Hydroxyl radical is highly reactive and directly attacks and damages biomolecules, whereas O_2_^•−^ contributes indirectly through secondary reactions that generate more toxic species such as ^•^OH and peroxynitrite (ONOO^−^) [3,4]. Lipids, especially polyunsaturated fatty acids, are susceptible to peroxidation leading to the formation of lipid hydroperoxides (LOOHs, an early lipid peroxidation marker), which are degraded to aldehydic byproducts such as malondialdehyde (MDA), considered among the most biotoxic late markers of lipid peroxidation. Malondialdehyde is extremely reactive and readily binds to proteins covalently, thereby altering their structure and functionality [3]. Proteins are also prone to oxidative modifications, one of them being carbonylation, which is an almost irreversible modification. Accumulation of carbonylated proteins can lead to cellular dysfunction [5].

Patients with end-stage kidney disease exhibit reduced life expectancy, mainly due to cardiovascular disease (CVD), in which OS has a critical role [6,7,8,9,10,11,12]. Extracorporeal circulation during hemodialysis can further increase OS, as reported to date, through factors such as filter membrane bio-incompatibility, antioxidant loss and anticoagulation [13,14,15].

Methodological limitations, heterogeneity in the design of previous studies as well as the non-specificity of the evaluated OS markers, have led to inconsistent and at times contradictory findings regarding the contribution of hemodialysis to OS. Specifically, blood O_2_^•−^ levels have been assessed by chemiluminescence (CL) of the non-specific probe lucigenin [16], which can also lead to overestimation due to redox cycling [17,18]. Many other studies have focused on the damage caused by ROS to biomolecules such as lipids, by using free MDA and thiobarbituric acid reactive substances (TBARSs) as markers of lipid peroxidation [19,20,21,22,23,24] (which may not accurately reflect biologically relevant oxidative damage), and glutathione (GSH) (measuring also its oxidized form, GSSG) [19,21,22,23,25,26]. Moreover, other studies have measured the activity of key antioxidant enzymes involved in ROS metabolism (superoxide dismutase, catalase, glutathione peroxidase, xanthine oxidase) [19,22,25,26,27,28], as well as natural antioxidants’ cumulative levels by the total antioxidant capacity (TAC) [28,29,30,31]. Even disease non-specific oxidized LDLs have been evaluated in patients on dialysis (by ELISA [28,31]). Therefore, the extent to which hemodialysis affects specific free radicals and downstream oxidative modifications remains unclear.

The present study addresses these limitations by employing highly specific methodologies and clinical markers that enable a more accurate assessment of blood plasma OS in the short-time frame of a single HD session, directly by certain ROS free radicals, and indirectly by oxidatively modified lipids (LOOHs, which represent early intermediates of lipid peroxidation and serve as sensitive indicators of acute oxidative changes) and proteins. Specifically, free radical O_2_^•−^ levels are measured before and after a single dialysis session, along with the peroxidized lipid product LOOHs, and protein-bound MDA, TBARSs, and also peroxidized protein products such as carbonyls (PrMDA, PrTBARSs, PrCOs). Additionally, these OS markers were also tested in relation to dialysis vintage, modality (low-flux conventional HD, high-flux conventional HD and pre-dilution HDF), comorbidities and medication.

## 2. Materials and Methods

### 2.1. Study Design and Patients

An observational pre-post dialysis study was conducted in 68 patients aged 25 to 86 years from the Hemodialysis Unit of the University Hospital of Patras, Greece, to assess changes in oxidative stress markers before and after a dialysis session. Demographic and clinical characteristics are shown in Table 1. Eligible participants were ESKD patients (>18 years) receiving regular hemodialysis (three times a week, 3–4 h/session) for at least 3 months. Exclusion criteria included systemic infection within 30 days prior to participation and active malignancy or malignancy within the previous 5 years. Inflammatory status was assessed using C-reactive protein (CRP) levels, anemia was assessed using hemoglobin (Hb) levels and serum albumin (ALB) was used as a marker of nutritional status. None of the included patients were receiving oral iron therapy and none had received any form of intravenous iron therapy within one month prior to enrollment. Hemodialysis was performed via an autologous arteriovenous fistula or graft. Patients undergoing hemodialysis via dialysis catheters were excluded from the study. All patients had residual urine output < 500 mL and therefore renal contribution to total clearance was not assessed. Patients were divided in three groups according to dialysis modality, i.e., those who received hemodialysis with low flux dialyzer (Fresenius-FX-10, Fresenius Medical Care, Bad Homburg, Germany; polysulfone membrane, effective surface area of 1.8 m^2^, ultrafiltration coefficient of 14 mL/h × mmHg), hemodialysis with high flux dialyzer (Fresenius-FX-80, Fresenius Medical Care, Bad Homburg, Germany; polysulfone membrane, effective surface area of 1.8 m^2^, ultrafiltration coefficient of 59 mL/h × mmHg) and pre-dilution hemodiafiltration (Fresenius-FX-80). All patients received an adequate dialysis dose as confirmed with a single-pool Kt/V of over 1.2 confirmed within a maximum of two weeks interval before the collection of blood samples for the analyses used in this study. Dialysate flow was set to a standard of 500 mL/min and replacement fluid volume for pre-dilution HDF was set to 20 L. Ultrafiltration volume per session ranged from 1500 to 3000 mL. All patients received anti-coagulation with a standard dose of 4000 IU of enoxaparin. All participants provided written informed consent. The study was conducted in accordance with the Declaration of Helsinki and approved by the Ethics Committee of the University Hospital of Patras (No. 316/04.09.2025).

### 2.2. Methods

Blood was collected into heparinized tubes immediately before initiation and after completion of the second weekly dialysis session, as a more representative patient’s state-condition and to avoid the long interdialytic interval.

#### 2.2.1. O_2_^•−^ Quantification

O_2_^•−^ was measured using hydroethidine (HE) as a probe at a final concentration of 25 μM, added to heparinized tubes prior to blood collection. Samples were incubated for 15 min at room temperature (RT) and centrifuged at 1500× *g* for 10 min to isolate plasma. Plasma samples were stored at −80 °C until analysis. A total of 0.1 mL of plasma was used for O_2_^•−^ quantification via measurement of 2-hydroxyethidium (2-OH-E^+^), a specific product of the reaction between O_2_^•−^ and HE, using High-Performance Thin Layer Chromatography [32]. O_2_^•−^ levels were expressed as pmol O_2_^•−^/mg protein.

#### 2.2.2. OS Markers Quantification

For the quantification of OS markers (LOOHs, PrMDA, PrTBARSs and PrCOs), blood was centrifuged at 1500× *g* for 10 min and plasma was protected from artificial oxidation by addition of the antioxidants BHA/BHT at final concentrations of 1/1 mM (using stock solution 200/200 mM in 100% ethanol). Plasma samples were stored at −80 °C until analysis. A total of 0.25 mL plasma was subjected to a fractionation protocol to isolate proteins and total lipids [33]. The protein fraction was analyzed for determination of PrMDA, PrTBARSs and PrCOs, while the lipid fraction was analyzed for the measurement of LOOHs. MDA and TBARSs were released from proteins by alkaline hydrolysis and measured after their reaction with thiobarbituric acid (TBA) under acidic conditions. PrMDA was quantified using fluorescence detection, while TBARSs were measured by photometric detection and both were expressed as pmol MDA/mg protein. The measurement of LOOHs is based on the reaction of Fe^3+^ (generated from the reaction of LOOH with Fe^2+^) with xylenol orange (XO) and the photometric quantification of the resulting XO–Fe complex [33]. LOOHs were expressed as nmol Cum-OOH equivalent/mg protein. PrCOs were quantified using a 2,4-dinitrophenylhydrazine (DNPH)-based photometric assay through the formation of protein carbonyl-DNPH hydrazones and were expressed as nmol carbonyls/mg protein [34].

For all assays, samples were measured in triplicate using three different dilutions, and mean values were calculated.

### 2.3. Statistical Analysis

Statistical analysis was performed using the IBM SPSS Statistics version 29.0.2.0 with the significance level set at 0.05. Normality of continuous variables was assessed using the Shapiro–Wilk test. Confidence intervals (95%) were calculated for parametric analyses, while non-parametric analyses are presented with effect sizes (r) and 95% bootstrap confidence intervals were applicable.

To evaluate OS marker levels before and after the hemodialysis session, paired *t*-tests (for O_2_^•−^, LOOH, PrMDA) or Wilcoxon signed-rank tests (for PrTBARSs, PrCO) were used, depending on the normality of paired differences.

Differences in OS markers among three dialysis modalities were evaluated using one-way ANOVA or the Kruskal–Wallis test, depending on data distribution and homogeneity of variances (assessed by Levene’s test). Post hoc Tukey analysis was performed to identify differences between groups. Analyses were based on both the pre-dialysis values of OS markers and their intradialytic changes (Δ = pre − post dialysis).

Spearman’s rank correlation was used for associations between dialysis vintage or patient’s age and each OS marker, due to non-normal distribution. Correlation coefficients (r) were reported as measures of effect size and 95% bootstrap confidence intervals (CIs) were calculated using 5000 resamples.

A robust linear regression using MM-estimation (R version 4.5.1; R Core Team, 2025; lmrob() function) was performed to assess whether the relationship between dialysis vintage and OS markers differs by dialysis modality.

Associations of OS markers with comorbidities, medication and sex were assessed with a Mann–Whitney U test.

No adjustment for multiple comparisons was performed.

Figures were generated using GraphPad Prism version 10.3.1.

## 3. Results

### 3.1. OS Biomarker Levels Before and After a Single Dialysis Session

**Figure 1:**
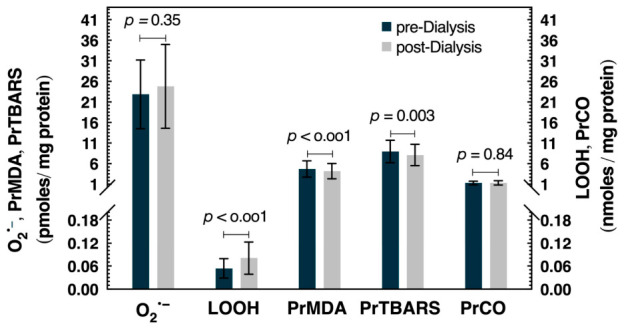
Effect of a single session of hemodialysis on ROS and OS markers in 68 patients. Data are presented as mean ± standard deviation (SD). Statistical analysis showed a significant increase for LOOH levels (by 1.5-fold, or 50%), while a significant decrease was observed for PrMDA (by 1.1-fold, or ~10%) and PrTBARSs (by 1.1-fold, or ~10%) levels. O_2_^•−^ and PrCO levels showed no significant difference.

Direct and indirect OS markers were assessed (Figure 1) in 68 hemodialysis patients (48 males, 20 females) with a median age of 65.5 years (IQR 55.18–73.07). The duration of hemodialysis ranged from 0.4 to 24.6 years, with a median of 6.15 years (IQR 2.5–9.03). Among participants, 47 were undergoing conventional hemodialysis (HD) and 21 were receiving pre-dilution hemodiafiltration (HDF).

O_2_^•−^ was used as a direct OS marker, while LOOHs, PrMDA, PrTBARSs and PrCOs were assessed as indirect markers in blood plasma obtained before and after the dialysis session. O_2_^•−^ levels were slightly elevated post-dialysis (22.86 ± 8.34 vs. 24.79 ± 10.2 pmoles O_2_^•−^/mg protein; mean difference = −1.93, 95% CI: −5.19 to 1.36, *p* = 0.35, Cohen’s d = −0.15), indicating a statistically non-significant change. However, the LOOH OS marker significantly increased (by 1.5-fold, or 50%) after the dialysis session (0.054 ± 0.025 vs. 0.081 ± 0.042 nmoles Cum-OOH equivalent/mg protein; mean difference = −0.027, 95% CI: −0.033 to −0.017, *p* < 0.001, Cohen’s d = −0.73), while the PrMDA and PrTBARS OS markers exhibited a modest but significant decrease (by 1.1-fold, or ~10%) post dialysis (PrMDA: 4.7 ± 1.98 vs. 4.21 ± 1.87 pmoles MDA/mg protein; mean difference = 0.49, 95% CI: 0.278 to 0.702, *p* < 0.001, Cohen’s d = 0.56, PrTBARS: 8.97 ± 2.74 vs. 8.12 ± 2.59 pmoles MDA/mg protein; Z = −2.94, *p* = 0.003, r = −0.36). Finally, the OS marker PrCO remained unchanged during dialysis (1.31 ± 0.43 and 1.33 ± 0.51 nmoles carbonyls/mg protein; Z = −0.2, *p* = 0.84, r = −0.02).

### 3.2. Impact of Dialysis Modalities on OS Biomarkers

To investigate the potential impact of dialysis modalities on OS burden, OS biomarkers’ levels were compared among patients undergoing low-flux conventional HD (N = 30), high-flux conventional HD (N = 17) and pre-dilution HDF (N = 21). Comparisons were performed both on pre-dialysis values and on intradialytic changes (Δ = value before dialysis − value after dialysis), to account for both baseline oxidative stress status and the effect of the dialysis itself. For pre-dialysis values, no significant differences were observed among the three modalities for O_2_^•−^ [one-way ANOVA: F(2, 65) = 0.16, *p* = 0.861, η^2^ = 0.005, 95% CI: 0.000 to 0.053], LOOH [Kruskal–Wallis: (H(2) = 5.408, *p* = 0.067, ε^2^ = 0.05), PrMDA [Κruskal-Wallis: (H(2) = 1.818, *p* = 0.403 ε^2^ = 0], PrTBARS [Kruskal–Wallis: (H(2) = 1.697, *p* = 0.428, ε^2^ = 0)] or PrCO [Kruskal–Wallis: (H(2) = 0.59, *p* = 0.745, ε^2^ = 0)] (Table 2). Similarly, no significant differences were found among modalities in intradialytic changes (Δ) for O_2_^•−^ [one-way ANOVA F(2, 65) = 1.99, *p* = 0.145, η^2^ = 0.058, 95% CI: 0.000 to 0.15)], PrMDA [one-way ANOVA F(2, 65) = 0.93, *p* = 0.4, η^2^ = 0.031, 95% CI: 0.000 to 0.133)], PrTBARS [Kruskal–Wallis: (H(2) = 0.152, *p* = 0.927, ε^2^ = 0)] or PrCOs [Kruskal–Wallis: (H(2) = 0.77, *p* = 0.681, ε^2^ = 0)] (Table 3). However, LOOH levels differed significantly among modalities, but only when comparisons were performed on Δ values [F(2, 65) = 4.434, *p* = 0.016, η^2^ =0.12, 95% CI: 0.02 to 0.24)]. Post hoc Tukey analysis revealed a significant difference between low-flux and high-flux HD (mean difference = −0.038, *p* = 0.021 95% CI: −0.07 to −0.005).

### 3.3. Associations Between OS Biomarkers and Dialysis Vintage

**Figure 2:**
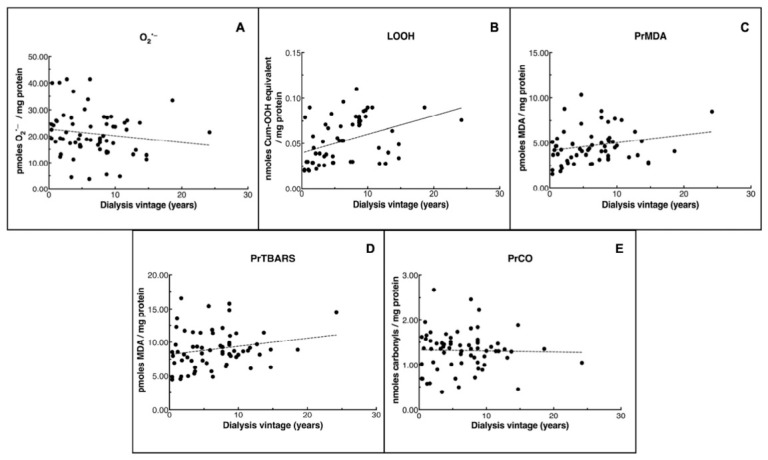
Spearman’s rank correlation tests between OS markers and dialysis vintage. Significant positive correlations were observed for markers such as LOOHs (r = 0.458, *p* = 0.005; (**B**)), PrMDA (r = 0.298, *p* = 0.018; (**C**)) and PrTBARSs (r = 0.272, *p* = 0.025; (**D**)). No significant correlations were found for O_2_^•−^ (r = −0.126, *p* = 0.47; (**A**)) and PrCOs (r = −0.112, *p* = 0.356; (**E**)).

To assess whether dialysis vintage correlates with the pre-dialysis levels of OS markers, Spearman’s rank correlation analyses were performed (Figure 2). A significant positive correlation was found between dialysis vintage and LOOHs (r = 0.458, *p* =0.005, 95% CI: 0.25 to 0.64; Figure 2B), PrMDA (r = 0.298, *p* = 0.018, 95% CI: 0.034 to 0.522; Figure 2C) and PrTBARSs (r = 0.272, *p* = 0.025, 95% CI: 0.026 to 0.484; Figure 2D). However, no significant correlation was observed between dialysis vintage and O_2_^•−^ (r = −0.126, *p* = 0.47, 95% CI: −0.456 to 0.216; Figure 2A) or PrCOs (r = −0.112, *p* = 0.356, 95% CI: −0.355 to 0.143; Figure 2E).

The same correlation analysis was applied to assess the relationship between patients’ age and OS markers. A significant positive correlation was observed between age and PrMDA. No significant correlations were found between age and the remaining OS markers. Detailed correlation coefficients are presented in Table 4.

Furthermore, robust linear regression analyses (MM-estimation) were performed to evaluate whether the slope of the relationship between pre-dialysis levels of each OS marker and dialysis vintage differs by dialysis modality. Low-flux HD was used as the reference category. Overall, no significant interaction effects were observed for O_2_^•−^ (high-flux HD: β = 0.999, *p* = 0.06; HDF: β = 0.274, *p* = 0.552), LOOHs (high-flux HD: β = 0.002, *p* = 0.208; HDF: β = −0.002, *p* = 0.708), PrMDA (high-flux HD: β = 0.066, *p* = 0.539; HDF: β = −0.032, *p* = 0.842), PrTBARSs (high-flux HD: β = −0.017, *p* = 0.886; HDF: β = −0.103, *p* = 0.832), and PrCOs (high-flux HD: β = −0.004, *p* = 0.917; HDF: β = −0.004, *p* = 0.904) (Table 5).

### 3.4. Additional Analyses

Finally, analyses were performed using the Mann–Whitney U tests to search for possible associations between the OS markers and patients’ clinical characteristics, specifically underlying comorbidities and medication. However, no significant differences were observed between OS markers and comorbidities such as Hypertension, CVD, CAD, PAD and Diabetes (Table 6).

Similarly, no significant associations were found between OS parameters and the use of medications including statins, alfacalcidol, paricalcitol, ACEis, ARBs, CaChBl, B blockers, Levocarnitine and Cholecalciferol (Table 7). Demographic characteristics such as sex were examined for potential associations with the oxidation markers and revealed no significant differences between males and females for any of the investigated OS markers (Table 8). In addition, Spearman’s rank correlation analyses were performed to assess associations between OS markers and laboratory parameters, including C-reactive protein (CRP), albumin (ALB) and hemoglobin (Hb). The corresponding results are presented in the Supplementary Materials (Table S1).

## 4. Discussion

In the present study, we evaluated five specific OS markers, including free radicals (O_2_^•−^) and oxidative modifications (LOOH, PrMDA, PrTBARS and PrCO) in blood plasma from patients undergoing maintenance dialysis before and after a single session. Our findings indicate that dialysis affects lipid and protein oxidation markers differently. O_2_^•−^ levels were slightly but not significantly increased following dialysis.

The most pronounced change among indirect OS markers was observed in LOOH levels, which increased 1.5-fold or by 50% after dialysis (Figure 1). Plasma LOOHs are found in oxidized lipoproteins, oxidized phospholipids and oxidized free fatty acids bound to albumin. The marked accumulation of LOOHs post-dialysis may reflect increased OS and/or a relative deficiency in plasma antioxidant defense. Potential mechanisms could include impaired scavenging of ROS (RO^•^ and ROO^•^) [35], alterations in plasma antioxidant systems, such as glutathione peroxidase 3 (GPx3), which has been reported to be reduced in CKD patients [36] or reduced availability of GSH due to dialysis-related loss [21,22,27,37]. However, as these parameters were not directly measured in the present study, these interpretations should be considered speculative.

Furthermore, PrMDA and PrTBARS levels showed a slight but significant decrease after dialysis by 1.1-fold, or ~10% (Figure 1). These markers reflect aldehydic products resulting from lipid peroxidation that are bound to proteins, providing a more accurate indication of the protein-related oxidative damage [38]. In contrast, free ΜDA and TBARSs do not represent the total levels produced by OS nor the fraction contributing to oxidative damage. It is also possible that the relatively short duration of a single dialysis session may not allow sufficient time for the formation of the late products of lipid peroxidation (MDA and TBARS) and their reaction with proteins. In addition, their partial removal during dialysis cannot be excluded.

PrCO levels remained unchanged during a single dialysis session, which is consistent with previous studies [22,27,28], and with the fact that the PrCO represent long term and mostly cumulative oxidative modifications [5].

Additionally, we compared low-flux HD, high-flux HD and pre-dilution HDF to assess potential differences in OS markers. No significant differences were observed among dialysis modalities for O_2_^•−^, PrMDA, PrTBARS, or PrCO, in pre-dialysis levels of OS markers (Table 2) or intradialytic changes (Δ) during a single session (Table 3). Significant differences were observed in LOOH between low-flux and high-flux HD only when analyzing the Δ values. However, given the sample size and subgroup distribution, these findings should be interpreted with caution. Although HDF has been associated with reduced OS in previous studies [28,39,40], no significant differences were detected in our study. Longitudinal measurements across multiple dialysis sessions may provide a more appropriate approach to reveal modality-related differences.

Next, we investigated whether dialysis vintage correlates with OS markers (Figure 2). Spearman’s rank analysis showed significant positive correlations between dialysis vintage and LOOHs, PrMDA and PrTBARSs, whereas no associations were found for O_2_^•−^ or PrCOs. Although these associations do not imply causality, they suggest a potential link between longer dialysis exposure and increased OS. Using the same analysis, no correlations were found between patient age and OS markers, except for PrMDA, which correlated positively with age (Table 4). Overall, these observations suggest that dialysis-related factors may have a stronger association with OS markers and underscore the need for further studies exploring the role of antioxidant defense and OS management strategies in HD patients, given its contribution to complications like CVDs, amyloidosis, inflammation and immune dysfunction [41].

Using robust linear regression with an interaction term, we tested whether the effect of dialysis vintage on OS markers differed by dialysis modality. No significant differences were found among patients receiving low-flux HD, high-flux HD or pre-dilution HDF. This may indicate that the increase in OS markers with dialysis vintage is influenced more by CKD-related factors and chronic exposure to dialysis treatment (Table 5).

Moreover, OS markers were analyzed for potential associations with patients’ comorbidities, medication and sex (Table 6, Table 7 and Table 8). Τhe comorbidities examined included hypertension, CAD, CVD, PAD and diabetes, which are common in HD patients and closely related to OS. Τhe medication included statin, alfacalcidol, paricalcitol, ACEi, ARBs, CaChBl, B-blocker, Levocarnitine, and Cholecalciferol. No statistically significant associations were observed. However, limited statistical power from small subgroups and medication adherence may have influenced the lack of significant findings.

Concluding, the findings of our study show that during hemodialysis LOOHs increased by 50%, as well as versus dialysis vintage (Figure 2). This may suggest that LOOHs reflect short-term changes in OS during a single hemodialysis session, as they are formed early in the lipid peroxidation cascade. In contrast, O_2_^•−^ levels were unchanged during the short period of hemodialysis. This free radical is predominantly generated intracellularly, in mitochondria, and is efficiently scavenged via its dismutation by H_2_O co-assisted by SOD [42]. Its presence does not necessarily reflect acute oxidative damage during an HD session. Rather, its indirect impact is mostly mediated through oxidative modifications on serum lipids and proteins. Furthermore, and in accordance with an increased LOOH-associated OS tendency are the levels of the lipid peroxidation markers PrMDA and PrTBARSs versus dialysis vintage (Figure 2), although they slightly decreased (by ~10%) during one hemodialysis session. On the other hand, the effect of hemodialysis on OS-associated protein damage is unchanged both after dialysis session as well as vintage. However, as we only measured the changes in oxidative stress markers before and after a single dialysis session, we cannot assess individual variability or long-term trends.

This study investigates the effect of a single dialysis session on OS, complementing previous research on OS during maintenance dialysis. Although limited by the sample size (68 subjects), the absence of longitudinal measurements and the lack of adjustment for multiple comparisons, which may increase the risk of type I error, our findings support the need for further investigation of OS in dialysis patients. Future studies with larger cohorts and repeated measurements over time, potentially including supplementation with dietary antioxidants are needed to better elucidate the long-term effects of dialysis and to evaluate targeted therapeutic interventions.

## Figures and Tables

**Table 1:** Demographic and clinical characteristics of study population.

		HD Patients
Age (years)		25 to 86
Median (IQR)		65.5 (55.18–73.07)
Dialysis vintage (years)		0.4 to 24.6
Median (IQR)		6.15 (2.5–9.03)
		N (%)
Sex		
Male		48 (70.6)
Female		20 (29.4)
Medical status		
Dialysis modality	Low-flux HD	30 (44.1)
	High-flux HD	17 (25)
	Pre-dilution HDF	21 (30.9)
Primary kidney disease		
Diabetic nephropathy		13 (19.1)
Hypertensive nephropathy		11 (16.2)
Glomerular disease		14 (20.6)
Polycystic kidney disease		3 (4.4)
Obstructive uropathy		5 (7.4)
Other		9 (13.2)
Unknown		13 (19.1)
Comorbidities		
Hypertension		31 (45.6%)
Cardiovascular disease (CVD)		30 (44.1%)
Coronary artery disease (CAD)		13 (19.1%)
Peripheral artery disease (PAD)		20 (29.4%)
Diabetes		24 (35.3%)
Medication		
Statin		22 (32.4)
Alfacalcidol		8 (11.8)
Paricalcitol		9 (13.2)
Angiotensin-converting enzyme inhibitors (ACEi)		3 (4.4)
Angiotensin receptor blockers (ARBs)		9 (13.2)
Calcium channel blockers (CaChBl)		19 (27.9)
B blockers		38 (55.9)
Levocarnitine		12 (17.6)
Cholecalciferol		17 (25)
Laboratory parameters		
C-reactive protein (mg/dL)		
Median (IQR)		0.61 (0.31–0.80)
Hemoglobin (g/dL)		
Median (IQR)		11.15 (10.27–11.6)
Albumin (g/dL)		
Median (IQR)		3.95 (3.88–4.13)

Note: The “Other” category includes nephrectomy-related cases, cardiorenal syndrome and cardiovascular disease.

**Table 2:** Comparison of three dialysis modalities based on pre-dialysis value of each OS marker.

OS Markers	Modality of Dialysis	N	Pre-Dialysis Values	*p* Value
O_2_^•−^	Low-flux HD	30	22.03 (±9.16)	0.861
	High-flux HD	17	23.56 (±6.8)	
	Pre-dilution HDF	21	23.08 (±11.22)	
LOOHs	Low-flux HD	30	0.045 (±0.027)	0.067
	High-flux HD	17	0.067 (±0.023)	
	Pre-dilution HDF	21	0.059 (±0.022)	
PrMDA	Low-flux HD	30	4.64 (±2.28)	0.403
	High-flux HD	17	5.19 (±1.93)	
	Pre-dilution HDF	21	4.42 (±1.59)	
PrTBARSs	Low-flux HD	30	8.55 (±2.78)	0.428
	High-flux HD	17	9.89 (±3.09)	
	Pre-dilution HDF	21	8.74 (±2.45)	
PrCOs	Low-flux HD	30	1.34 (±0.51)	0.745
	High-flux HD	17	1.34 (±0.42)	
	Pre-dilution HDF	21	1.24 (±0.36)	

Νotes: values are presented as mean ± standard deviation (SD). Abbreviations: HD, hemodialysis; HDF, hemodiafiltration; O_2_^•−^, superoxide radical; LOOHs, lipid peroxides; PrMDA, protein bound-malondialdehyde; PrTBARSs, protein bound-thiobarbituric acid reactive substances; PrCOs, protein carbonyls.

**Table 3:** Comparison of three dialysis modalities based on dialysis-induced difference (Δ-value = pre − post dialysis) of each OS marker.

OS Markers	Modality of Dialysis	N	Δ-Value (Pre − Post Dialysis)	*p* Value
O_2_^•−^	Low-flux HD	30	−2.3 (±2.4)	0.145
	High-flux HD	17	−1.6 (±2.9)	
	Pre-dilution HDF	21	−2.1 (±2.6)	
LOOHs	Low-flux HD	30	−0.045 (±0.031)	0.016
	High-flux HD	17	−0.008 (±0.035)	
	Pre-dilution HDF	21	−0.018 (±0.029)	
PrMDA	Low-flux HD	30	0.63 (±0.7)	0.4
	High-flux HD	17	0.62 (±0.81)	
	Pre-dilution HDF	21	0.31 (±1.05)	
PrTBARSs	Low-flux HD	30	0.77 (±3.07)	0.927
	High-flux HD	17	1.28 (±2.04)	
	Pre-dilution HDF	21	0.77 (±1.92)	
PrCOs	Low-flux HD	30	−0.06 (±0.31)	0.681
	High-flux HD	17	0.02 (±0.27)	
	Pre-dilution HDF	21	0.03 (±0.22)	

Notes: values are presented as mean ± standard deviation (SD). Abbreviations: HD, hemodialysis; HDF, hemodiafiltration; O_2_^•−^, superoxide radical; LOOHs, lipid peroxides; PrMDA, protein bound-malondialdehyde; PrTBARSs, protein bound-thiobarbituric acid reactive substances; PrCOs, protein carbonyls.

**Table 4:** Correlation of OS markers and patients age using Spearman’s rho coefficient (r).

OS Markers vs. Age	Spearman’s rho Coefficient (r)	*p* Value	95% CI
O_2_^•−^	−0.006	0.973	−0.344–0.23
LOOHs	−0.121	0.483	−0.449–0.25
PrMDA	0.303	0.016	0.059–0.519
PrTBARSs	0.187	0.127	−0.053–0.409
PrCOs	0.204	0.09	−0.027–0.415

**Table 5:** Associations between dialysis parameters (modality, vintage) and OS markers using robust linear regression.

Dependent Variable					
O_2_^•−^ pre-dialysis	Estimate (β)	Std. Error	t value	95% CI	*p*-value
Intercept (reference: low-flux HD)	24.012	2.803	8.567	18.29 to 29.74	1.47 × 10^−9^ ***
Dialysis vintage	−0.37	0.332	−1.113	−1.048 to 0.31	0.275
Modality_high-flux HD (difference vs. low-flux HD)	−5.846	4.65	−1.257	−15.34 to 3.65	0.218
Modality_HDF (difference vs. low-flux HD)	−0.489	5.875	−0.083	−12.49 to 11.51	0.934
Dialysis vintage:Modality_high-flux HD (interaction)	0.999	0.51	1.959	−0.043 to 2.042	0.06
Dialysis vintage:Modality_HDF (interaction)	0.274	0.456	0.601	−0.66 to 1.21	0.552
Observations	68				
R^2^	0.071				
Adjusted R^2^	−0.083				
Residual Std. Error	8.013 (df = 62)				
LOOH pre-dialysis	Estimate (β)	Std. Error	t value	95% CI	*p*-value
Intercept (reference: low-flux HD)	0.034	0.011	2.899	0.01 to 0.06	0.007 ***
Dialysis vintage	0.001	0.0008	1.125	−0.001 to 0.003	0.270
Modality_high-flux HD (difference vs. low-flux HD)	0.012	0.014	0.825	−0.018 to 0.042	0.416
Modality_HDF (difference vs. low-flux HD)	0.027	0.021	1.269	−0.017 to 0.07	0.215
Dialysis vintage:Modality_high-flux HD (interaction)	0.002	0.001	1.288	−0.001 to 0.004	0.208
Dialysis vintage:Modality_HDF (interaction)	−0.002	0.005	−0.379	−0.01 to 0.008	0.708
Observations	68				
R^2^	0.32				
Adjusted R^2^	0.199				
Residual Std. Error	0.02 (df = 62)				
PrMDA pre-dialysis	Estimate (β)	Std. Error	t value	95% CI	*p*-value
Intercept (reference: low-flux HD)	3.324	0.769	4.324	1.75 to 4.9	0.001 ***
Dialysis vintage	0.025	0.082	0.302	−0.14 to 0.19	0.765
Modality_high-flux HD (difference vs. low-flux HD)	−0.069	0.994	−0.070	−2.1 to 1.97	0.945
Modality_HDF (difference vs. low-flux HD)	0.295	0.925	0.319	−1.6 to 2.19	0.752
Dialysis vintage:Modality_high-flux HD (interaction)	0.066	0.105	0.623	−0.15 to 0.28	0.539
Dialysis vintage:Modality_HDF (interaction)	−0.032	0.161	−0.201	−0.36 to 0.3	0.842
Observations	68				
R^2^	0.095				
Adjusted R^2^	−0.066				
Residual Std. Error	1.656 (df = 62)				
PrTBARS pre-dialysis	Estimate (β)	Std. Error	t value	95% CI	*p*-value
Intercept (reference: low-flux HD)	6.939	1.037	6.693	4.81 to 9.06	2.9 × 10^−7^ ***
Dialysis vintage	0.112	0.121	0.932	−0.13 to 0.36	0.359
Modality_high-flux HD (difference vs. low-flux HD)	0.351	1.13	0.310	−1.96 to 2.66	0.759
Modality_HDF (difference vs. low-flux HD)	0.428	1.775	0.241	−3.21 to 4.06	0.811
Dialysis vintage:Modality_high-flux HD (interaction)	−0.017	0.122	−0.145	−0.27 to 0.23	0.886
Dialysis vintage:Modality_HDF (interaction)	−0.103	0.481	−0.214	−1.09 to 0.88	0.832
Observations	68				
R^2^	0.06				
Adjusted R^2^	−0.107				
Residual Std. Error	2.282 (df = 62)				
PrCO pre-dialysis	Estimate (β)	Std. Error	t value	95% CI	*p*-value
Intercept (reference: low-flux HD)	1.104	0.231	4.789	0.63 to 1.57	4.22 × 10^−5^ ***
Dialysis vintage	0.004	0.032	0.144	−0.06 to 0.07	0.887
Modality_high-flux HD (difference vs. low-flux HD)	0.036	0.307	0.116	−0.59 to 0.66	0.908
Modality_HDF (difference vs. low-flux HD)	−0.027	0.273	−0.099	−0.58 to 0.53	0.922
Dialysis vintage:Modality_high-flux HD (interaction)	−0.004	0.036	−0.105	−0.08 to 0.07	0.917
Dialysis vintage:Modality_HDF (interaction)	−0.004	0.033	−0.122	−0.07 to 0.06	0.904
Observations	68				
R^2^	0.006				
Adjusted R^2^	−0.16				
Residual Std. Error	0.471 (df = 62)				

Note: Reference category: low-flux HD. Dialysis vintage is expressed in years. Estimates represent regression coefficients derived from robust linear regression (MM-estimation, lmrob function), which is less sensitive to outliers. Interaction terms indicate effect modification, reflecting differences in the slope of association between dialysis vintage and OS markers across modalities (high-flux HD, HDF) relative to the reference group (low-flux HD). Model fit indices (R^2^ and adjusted R^2^) are reported for descriptive purposes. *p*-values < 0.05 were considered statistically significant. *** *p* < 0.001.

**Table 6:** Underlying comorbidities and OS markers in dialysis patients.

	OS Markers	Patients	N	Value	Effect Size r	*p*-Value
Hypertension	O_2_^•−^	with Hypertension	31	23.24 (±9.88)	r = −0.04	0.74
		no Hypertension	37	22.54 (±7.28)		
	LOOHs	with Hypertension	31	0.058 (±0.026)	r = −0.119	0.326
		no Hypertension	37	0.051 (±0.026)		
	PrMDA	with Hypertension	31	4.44 (±1.79)	r = −0.07	0.548
		no Hypertension	37	4.76 (±2.17)		
	PrTBARSs	with Hypertension	31	8.58 (±2.68)	r = −0.12	0.397
		no Hypertension	37	9.32 (±3.06)		
	PrCOs	with Hypertension	31	1.19 (±0.48)	r = −0.22	0.062
		no Hypertension	37	1.40 (±0.39)		
CVD	O_2_^•−^	with CVD	30	23.34 (±7.76)	r = −0.03	0.805
		no CVD	38	22.48 (±9.5)		
	LOOHs	with CVD	30	0.053 (±0.024)	r = −0.046	0.702
		no CVD	38	0.056 (±0.027)		
	PrMDA	with CVD	30	4.85 (±1.69)	r = −0.15	0.212
		no CVD	38	4.56 (±2.1)		
	PrTBARSs	with CVD	30	9.2 (±2.48)	r = −0.11	0.361
		no CVD	38	8.78 (±3.01)		
	PrCOs	with CVD	30	1.43 (±0.41)	r = −0.22	0.075
		no CVD	38	1.2 (±0.44)		
CAD	O_2_^•−^	with CAD	13	21.46 (±10.7)	r = −0.13	0.29
		no CAD	55	23.17 (±8.5)		
	LOOHs	with CAD	13	0.048 (±0.027)	r = −0.13	0.283
		no CAD	55	0.056 (±0.026)		
	PrMDA	with CAD	13	4.61 (±1.5)	r = −0.048	0.693
		no CAD	55	4.71 (±2.06)		
	PrTBARSs	with CAD	13	9.0 (±2.29)	r = −0.04	0.727
		no CAD	55	8.95 (±2.9)		
	PrCOs	with CAD	13	1.38 (±0.4)	r = −0.08	0.493
		no CAD	55	1.29 (±0.45)		
PAD	O_2_^•−^	with PAD	20	24.12 (±6.03)	r = −0.09	0.26
		no PAD	48	22.34 (±9.21)		
	LOOHs	with PAD	20	0.059 (±0.023)	r = −0.089	0.462
		no PAD	48	0.054 (±0.027)		
	PrMDA	with PAD	20	5.11 (±1.76)	r = −0.21	0.09
		no PAD	48	4.51 (±1.96)		
	PrTBARSs	with PAD	20	9.74 (±2.23)	r = −0.23	0.06
		no PAD	48	8.64 (±2.94)		
	PrCOs	with PAD	20	1.37 (±0.29)	r = −0.11	0.367
		no PAD	48	1.28 (±0.49)		
Diabetes	O_2_^•−^	with Diabetes	24	20.34 (±8.77)	r = −0.016	0.055
		no Diabetes	44	24.1 (±8.81)		
	LOOHs	with Diabetes	24	0.053 (±0.026)	r = −0.049	0.683
		no Diabetes	44	0.056 (±0.026)		
	PrMDA	with Diabetes	24	4.77 (±1.81)	r = −0.097	0.416
		no Diabetes	44	4.50 (±1.97)		
	PrTBARSs	with Diabetes	24	9.42 (±2.49)	r = −0.17	0.158
		no Diabetes	44	8.72 (±2.92)		
	PrCOs	with Diabetes	24	1.36 (±0.36)	r = −0.18	0.147
		no Diabetes	44	1.27 (±0.47)		

Notes: values are presented as mean (M) ± standard deviation (SD). Effect size r is calculated as r = Z/√N. O_2_^•−^ levels are expressed as pmoles O_2_^•−^/mg protein, LOOH levels are expressed as nmoles Cum-OOH equivalent/mg protein, PrMDA and PrTBARS levels are expressed as pmoles MDA/mg protein and PrCO levels are expressed as nmoles carbonyls/mg protein.

**Table 7:** Medication and OS markers in dialysis patients.

	OS Markers	Patients	N	Value	Effect Size r	*p*-Value
Statin	O_2_^•−^	TM	22	20.8 (±9.82)	r = −0.13	0.281
		NTM	46	23.6 (±8.53)		
	LOOHs	TM	22	0.05 (±0.21)	r = −0.11	0.379
		NTM	46	0.057 (±0.27)		
	PrMDA	TM	22	5.12 (±1.95)	r = −0.175	0.149
		NTM	46	4.49 (±1.88)		
	PrTBARSs	TM	22	9.22 (±2.72)	r = −0.04	0.714
		NTM	46	8.84 (±2.83)		
	PrCOs	TM	22	1.26 (±0.39)	r = −0.08	0.504
		NTM	46	1.33 (±0.46)		
Alfacalcidol	O_2_^•−^	TM	8	24.68 (±12.53)	r = −0.01	0.945
		NTM	60	22.65 (±8.53)		
	LOOHs	TM	8	0.045 (±0.006)	r = −0.09	0.442
		NTM	60	0.056 (±0.027)		
	PrMDA	TM	8	3.77 (±0.98)	r = −0.16	0.183
		NTM	60	4.81 (±1.97)		
	PrTBARSs	TM	8	7.8 (±1.58)	r = −0.13	0.278
		NTM	60	9.12 (±2.87)		
	PrCOs	TM	8	1.27 (±0.48)	r = −0.014	0.909
		NTM	60	1.31 (±0.44)		
Paricalcitol	O_2_^•−^	TM	9	24.05 (±4.97)	r = −0.1	0.39
		NTM	59	22.64 (±9.43)		
	LOOHs	TM	9	0.065 (±0.26)	r = −0.15	0.218
		NTM	59	0.054 (±0.026)		
	PrMDA	TM	9	4.62 (±1.49)	r = −0.027	0.821
		NTM	59	4.7 (±1.97)		
	PrTBARSs	TM	9	9.29 (±2.52)	r = −0.07	0.581
		NTM	59	8.92 (±2.88)		
	PrCOs	TM	9	1.25 (±0.39)	r = −0.06	0.634
		NTM	59	1.31 (±0.45)		
ACEi	O_2_^•−^	TM	3	22.89 (±3.9)	r = −0.03	0.802
		NTM	65	22.86 (±9.06)		
	LOOHs	TM	3	0.065 (±0.006)	r = −0.15	0.206
		NTM	65	0.053 (±0.025)		
	PrMDA	TM	3	5.68 (±1.98)	r = −0.13	0.276
		NTM	65	4.64 (±1.91)		
	PrTBARSs	TM	3	11.28 (±2.53)	r = −0.18	0.143
		NTM	65	8.85 (±2.76)		
	PrCOs	TM	3	0.93 (±0.33)	r = −0.22	0.073
		NTM	65	1.32 (±0.44)		
ARB	O_2_^•−^	TM	9	29.63 (±11.57)	r = −0.22	0.096
		NTM	59	21.84 (±8.08)		
	LOOHs	TM	9	0.065 (±0.11)	r = −0.22	0.066
		NTM	59	0.052 (±0.026)		
	PrMDA	TM	9	4.13 (±0.97)	r = −0.07	0.544
		NTM	59	4.77 (±2.0)		
	PrTBARSs	TM	9	8.1 (±1.74)	r = −0.12	0.307
		NTM	59	9.1 (±2.89)		
	PrCOs	TM	9	1.24 (±0.37)	r = −0.03	0.835
		NTM	59	1.32 (±0.45)		
CaChBl	O_2_^•−^	TM	19	24.18 (±9.72)	r = −0.16	0.194
		NTM	49	22.09 (±8.39)		
	LOOHs	TM	19	0.053 (±0.032)	r = −0.126	0.299
		NTM	49	0.056 (±0.022)		
	PrMDA	TM	19	4.53 (±1.9)	r = −0.01	0.94
		NTM	49	4.75 (±1.93)		
	PrTBARSs	TM	19	9.03 (±2.95)	r = −0.02	0.897
		NTM	49	8.94 (±2.74)		
	PrCOs	TM	19	1.19 (±0.56)	r = −0.18	0.144
		NTM	49	1.35 (±0.38)		
B blockers	O_2_^•−^	TM	38	22.99 (±10.21)	r = −0.03	0.84
		NTM	30	22.66 (±6.51)		
	LOOHs	TM	38	0.055 (±0.025)	r = −0.09	0.466
		NTM	30	0.055 (±0.027)		
	PrMDA	TM	38	4.57 (±1.85)	r = −0.076	0.529
		NTM	30	4.84 (±2.02)		
	PrTBARSs	TM	38	8.68 (±2.78)	r = −0.11	0.38
		NTM	30	9.33 (±2.77)		
	PrCOs	TM	38	1.32 (±0.52)	r = −0.01	0.951
		NTM	30	1.29 (±0.32)		
Levocarnitine	O_2_^•−^	TM	12	17.1 (±9.35)	r = −0.17	0.16
		NTM	56	23.54 (±8.67)		
	LOOHs	TM	12	0.06 (±0.014)	r = −0.077	0.527
		NTM	56	0.055 (±0.026)		
	PrMDA	TM	12	5.24 (±1.91)	r = −0.146	0.228
		NTM	56	4.57 (±1.9)		
	PrTBARSs	TM	12	9.01 (±2.92)	r = −0.002	0.987
		NTM	56	8.96 (±2.77)		
	PrCOs	TM	12	1.43 (±0.46)	r = −0.1	0.398
		NTM	56	1.28 (±0.44)		
Cholecalciferol	O_2_^•−^	TM	17	20.43 (±4.4)	r = −0.25	0.118
		NTM	51	23.67 (±9.42)		
	LOOHs	TM	17	0.051 (±0.029)	r = −0.09	0.481
		NTM	51	0.056 (±0.025)		
	PrMDA	TM	17	5.36 (±2.21)	r = −0.147	0.226
		NTM	51	4.47 (±1.76)		
	PrTBARSs	TM	17	9.39 (±3.12)	r = −0.06	0.645
		NTM	51	8.82 (±2.67)		
	PrCOs	TM	17	1.31 (±0.3)	r = −0.01	0.938
		NTM	51	1.30 (±0.49)		

Notes: values are presented as mean (M) ± standard deviation (SD). Effect size r is calculated as r = Z/√N. TK refers to patients taking medication, while NTM refers to non-medicated patients. O_2_^•−^ levels are expressed as pmoles O_2_^•−^/mg protein, LOOH levels are expressed as nmoles Cum-OOH equivalent/mg protein, PrMDA and PrTBARS levels are expressed as pmoles MDA/mg protein and PrCO levels are expressed as nmoles carbonyls/mg protein.

**Table 8:** Comparison of OS markers between male and female dialysis patients using Mann–Whitney U test.

	OS Markers	Patients	N	Value	Effect Size r	*p*-Value
Sex	O_2_^•−^	Male	48	23.87 ± 8.63	r = −0.168	0.166
		Female	20	20.43 ± 8.84		
	LOOHs	Male	48	0.056 ± 0.027	r = −0.142	0.242
		Female	20	0.05 ± 0.02		
	PrMDA	Male	48	4.44 ± 1.67	r = −0.09	0.459
		Female	20	5.32 ± 2.52		
	PrTBARSs	Male	48	8.63 ± 2.45	r = −0.169	0.163
		Female	20	10.07 ± 3.48		
	PrCOs	Male	48	1.24 ± 0.4	r = −0.21	0.082
		Female	20	1.48 ± 0.46		

Notes: values are presented as mean (M) ± standard deviation (SD). Effect size r is calculated as r = Z/√N. O_2_^•−^ levels are expressed as pmoles O_2_^•−^/mg protein, LOOH levels are expressed as nmoles Cum-OOH equivalent/mg protein, PrMDA and PrTBARS levels are expressed as pmoles MDA/mg protein and PrCO levels are expressed as nmoles carbonyls/mg protein.

## Data Availability

The data underlying this article will be shared on reasonable request to the corresponding author.

## Supplementary Materials

The following supporting information can be downloaded at: https://www.mdpi.com/article/10.3390/cimb48050482/s1, Table S1: Correlation of OS markers and laboratory parameters using Spearman’s rho coefficient (r).
