# Supplementary material for: Impact of a Single Hemodialysis Session on Oxidative Stress-Inducing and Oxidative Damage Biomarkers in End-Stage Kidney Disease Patients
Source: Curr Issues Mol Biol. 2026 May 6;48(5):482. doi: 10.3390/cimb48050482 (PMC13204637; doi:10.3390/cimb48050482)
Supplement: Supplementary file 1 [file cimb-48-00482-s001.zip › cimb-4252041-supplementary.pdf]

Supplementary material

Table S1: Correlation of OS markers and laboratory parameters using Spearman's rho coefficient (r).

		**Spearman's rho (r)**	**P-value**	**95 % CI**
	**$O_2^{\bullet -}$**	0.104	0.535	-0.24 to 0.45
	**LOOH**	0.206	0.216	-0.13 to 0.5
CRP	**PrMDA**	0.244	0.067	-0.026 to 0.48
	**PrTBARS**	0.163	0.21	-0.11 to 0.4
	**PrCO**	0.108	0.40	-0.16 to 0.35
	**$O_2^{\bullet -}$**	-0.09	0.591	-0.43 to 0.26
	**LOOH**	0.298	0.069	-0.005 to 0.55
Hb	**PrMDA**	-0.037	0.785	-0.31 to 0.24
	**PrTBARS**	0.003	0.981	-0.25 to 0.28
	**PrCO**	0.062	0.631	-0.19 to 0.32
	**$O_2^{\bullet -}$**	-0.078	0.642	-.038 to 0.27
	**LOOH**	0.121	0.468	-0.19 to 0.42
ALB	**PrMDA**	0.13	0.336	-0.14 to 0.4
	**PrTBARS**	-0.026	0.845	-0.29 to 0.24
	**PrCO**	-0.079	0.544	-0.3 to 0.16

Note: Correlation coefficients (r) were reported as measures of effect size and 95 % bootstrap confidence intervals (CI) were calculated using 5000 resamples. CRP: C-reactive protein; ALB: Albumin; Hb: Hemoglobin
